# Monolithic III–V on Silicon Plasmonic Nanolaser Structure for Optical Interconnects

**DOI:** 10.1038/srep14067

**Published:** 2015-09-15

**Authors:** Ning Li, Ke Liu, Volker J. Sorger, Devendra K. Sadana

**Affiliations:** 1 IBM T. J. Watson Research Center, 1101 Kitchawan Road, Yorktown Heights, New York 10598, USA; 2 Department of Electrical and Computer Engineering, George Washington University, Washington, DC 20052, USA; 3 The Key Laboratory of Optoelectronics Technology, Ministry of Education, Beijing University of Technology, Beijing 100124, P.R. China

## Abstract

Monolithic integration of III–V semiconductor lasers with Si circuits can reduce cost and enhance performance for optical interconnects dramatically. We propose and investigate plasmonic III–V nanolasers as monolithically integrated light source on Si chips due to many advantages. First, these III–V plasmonic light sources can be directly grown on Si substrates free of crystallographic defects due to the submicron cavity footprint (250 nm × 250 nm) being smaller than the average defect free region size of the heteroepitaxial III–V material on Si. Secondly, the small lateral and vertical dimensions facilitate process co-integration with Si complementary metal-oxide-semiconductor (CMOS) in the front end of the line. Thirdly, combining with monolithically integrated CMOS circuits with low device capacitance and parasitic capacitance, the nano-cavity optoelectronic devices consume orders of magnitude less power than the conventional lasers and reduce the energy consumption. Fourthly, the modulation bandwidth of the plasmonic light-sources is enhanced to significantly higher than conventional lasers due to enhanced photon state density and transition rate. In addition, we show that these device performance are very robust after taking into account the surface recombination and variations in device fabrication processes.

An on-chip light source is preferred over being off-chip for optical interconnects for various reasons^1^,^2^; 1) the power loss from couplers and splitters is saved for on-chip sources. 2) on-chip laser can be directly modulated, thus eliminating the need to deploy modulators. Among the on-chip laser integration approaches, a monolithically grown laser on Si has many advantages over hybrid bonding. For instance the process can be simplified and allows for more design flexibility, much lower packaging cost, and most importantly, smaller parasitic device capacitance. Moreover, synergies can be explored between such nanoscale photonic elements and their electronic counterparts, as metal-oxide-semiconductor field-effect transistors that use III–V semiconductors as channel materials are being monolithically integrated on Si as next generation logic devices^3^.

While III–V lasers monolithically grown on Si were demonstrated decades ago, the prevailing challenge of these devices remains the short laser lifetimes due to a high defect density in the heteroepitaxial material^4^,^5^,^6^. Because of polarity difference, large lattice and thermal expansion mismatch, the crystallographic defect density of III–V materials grown on Si is several orders of magnitude higher than the same material grown on lattice-matched III–V substrates. The typical threading dislocation density of III–Vs grown on Si is ~10^8^ cm^−2^ 7, which corresponds to ~1 defect per 1 μm^2^. Since conventional III–V edge emitting lasers and vertical cavity surface emitting lasers (VCSELs) have dimensions of at least tens of microns, when growing on Si directly, these devices contain a significant number of crystallographic defects. The reaction and growth of these defects during laser operation is the cause of the device failure and short laser lifetime^8^. However, if the laser’s dimension is reduced below the average spacing between two adjacent defects, the device can be made defect free with a high probability. Here we investigate plamonic lasers due to their sub-diffraction limited dimensions, smallest among all nanolasers^9^,^10^.

Besides the advantage of small cavity size, monolithic integration of nanolasers on Si can also dramatically reduce energy consumption of the networking fabric in large-scale computing and data centers. This is because nanometer-scale lasers and photodetectors not only have a lower capacitance, but also feature unique internal physical effects, which allow for a more efficient photon utilization inside the gain material. Together both effects point towards the potential to significantly lower the power consumption compared to current technology options^11^. In addition, highly scaled nano-cavity plasmonic lasers further enhance the modulation speed, and can have superior frequency response thus achieving much higher direct modulation speed than other light sources. Growing III–V nano-photonics components on Si monolithically in conjunction with Si and III-V electronics will eventually meet the requirement of 100 s of GHz bandwidth and fJs/bit energy efficiency for the ever growing optical data transmission demands in data centers and high performance computers.

## Results

**Figure 1:**
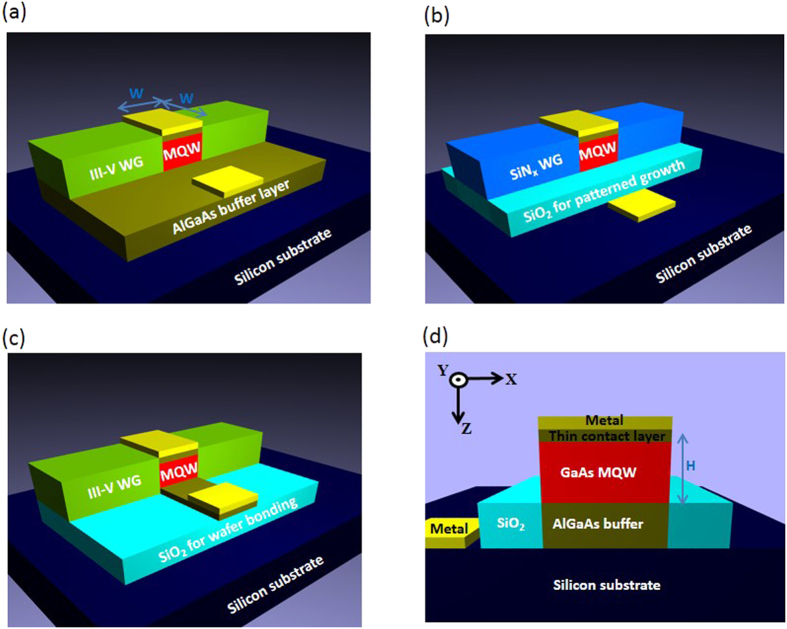
Schematic structures of III–V plasmonic nanolasers integrated on Si substrate: (**a**) Angled view of plasmoinc nanolasers monolithically grown on Si with blanket AlGaAs buffer layer. (**b**) Plasmoinc nanolasers monolithically grown on Si with selective growth in oxide confined pattern. (**c**) Plasmoinc nanolasers bonded to Si substrate using oxide wafer bonding. (**d**) Cross-section layout of the monolithic plasmonic nanolaser shown in (**b**).

Because of the aforementioned reasons, we investigated three nanolaser configurations (Fig. 1), including two monolithic device structures and one hybrid bonded device structure in comparison. In the first monolithic approach, the laser structure is grown on Si with a blanket Al_0.6_Ga_0.4_As buffer layer, where the waveguide core consists of Al_0.3_Ga_0.7_As (Fig. 1a). In the second monolithic approach, the laser structure is monolithically grown on the Si substrate via a selective growth in a hole patterned on a SiO_2_ mask layer (Fig. 1b). Only the laser cavity region is grown on the substrate on the Al_0.6_Ga_0.4_As buffer layer. Here the waveguide material is chosen to be of SiN_x_. In the hybrid bonding approach, the laser structure is first grown on a lattice-matched GaAs substrate and then transferred to the Si substrate via oxide bonding (Fig. 1c). All these devices use the same structure of the plasmonic laser active region. For example, the cross section of the monolithic structure with selective growth is shown in Fig. 1d. The active regions of all these plasmonic nano-cavities consist of GaAs (10 nm)/Al_0.3_Ga_0.7_As (10 nm) multiple quantum wells (MQW). The nanolaser cavity is created by depositing a metal layer atop the stack to form a plasmonic ‘island’, which features a dielectrically-loaded surface plasmon polariton (DLSPP) mode^12^. A 20 nm-thick highly doped p^+^ GaAs contact layer is inserted between the MQW and the metal, serving as the top Ohmic contact layer for the electrically pumped laser diode. All the metal-cavity has a square shape with cavity width of *W *= 250 nm. Although Au is used here as the metal material, other metals such as silver, aluminum, or copper can be used instead to be more compatible with Si complementary metal-oxide-semiconductor (CMOS). The second electrode can be formed using the bottom n^+^ layer on the side of the device. For the bonded device, a thin highly doped layer is used for making the bottom side contact. The details of the simulation approach are described in the methods section of the paper.

### Defect density consideration

The sub-diffraction limited device footprint (250 nm × 250 nm) of the nanolaser explored here is 1/16 the size of the average defect free region in the III–V material on Si given a high defect density of ~10^8^ cm^−2^. Assuming uniform distribution of defects, this results in a defect-free yield of 94% for these devices. With advanced growth techniques such as relaxed Ge buffer layers, the defect density of III–V on Si can be reduced to ~10^6^ cm^−2^ 7,8. In this case, our nanolaser has a dimension that is 1/1600 the size of the average defect free region. The defect-free yield of our nanolaser is >99.9% in this case compared to zero defect free device in the conventional monolithic III–V laser on Si case. In addition, the selective growth structure design is compatible with the aspect ratio trapping (ART) growth technique^13^,^14^, where threading dislocations are terminated at the oxide side walls and result in defect free regions. Due to the high aspect ratio required in the ART growth, the defect-free growth region is typically only a few hundred nanometers wide. Therefore, in order to make lasers in this region, the laser dimensions need to be reduced to hundreds of nanometer scale, as designed in this work.

### Quality factor and Purcell factor

**Figure 2:**
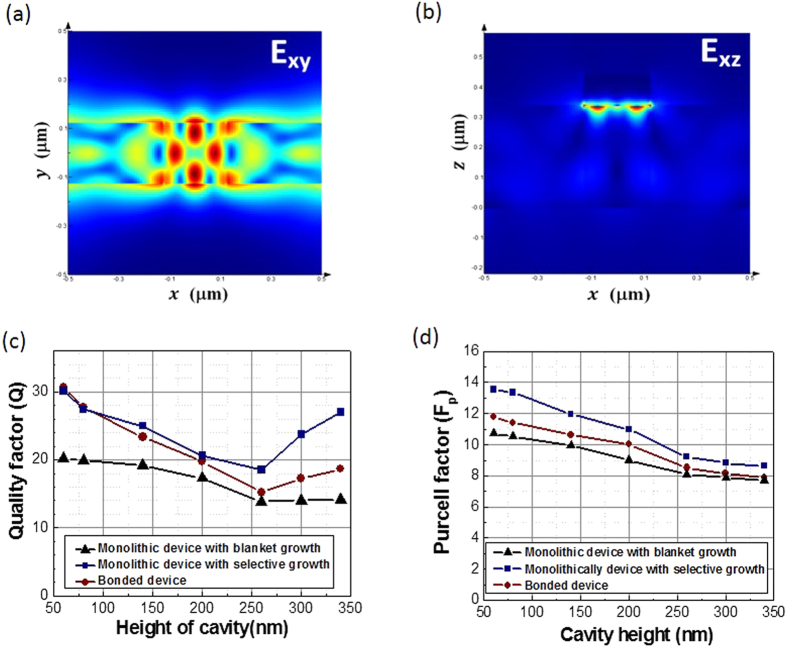
Simulated cavity properties by FDTD simulation: (**a**) Electric field profile in the X–Y plane in the middle of the cavity and waveguide. (**b**) Electric field profile in the X–Z plane at the center of the cavity. (**c**) Cavity quality factor (*Q*) as a function of the height of the cavity (*H*). (**d**) Purcell factor (*F*_*p*_) as a function of *H*.

Towards comparing the performance for three different nanolaser configurations, cavity quality factor *Q* and Purcell factor *F*_*p*_ are two important parameters for the plasmonic nanolaser design. All the optical simulation of these lasers is performed using commercially available finite difference time domain (FDTD) software, as described in the Methods section. The *Q* factors were calculated for all the modes where the mode of highest *Q* was selected for a target lasing wavelength of ~850 nm. As an example, the mode profile of the monolithic device with blanket growth is plotted in the x–y cross section (Fig. 2a) and x–z cross section (Fig. 2b), respectively. While the lateral dimension of the cavity is fixed at 250 nm × 250 nm, the height of the cavity (*H*) is swept to find the optimal design. It is straightforward that the monolithic device grown on Si in the oxide pattern exhibits highest *Q* and *F*_*p*_, due to better light confinement originating from the large index contrast between the high refractive index GaAs/AlGaAs cavity region and the SiN_x_/SiO_2_ waveguides (Fig. 2c). However, the differences of *Q* among three devices are rather small, because the plasmonic mode for all devices is mainly concentrated at the top interface between metal and dielectric material (Fig. 2b), which is not affected much by the materials on the bottom and side of the cavity.

The Purcell factor *F*_*p*_ is proportional to *Q/V*_*mod*_, where *V*_*mod*_ is the effective volume of an optical mode in the cavity. In our design the cavity mode volume of the devices here is ~0.9 times of the diffraction limit size *(λ/2n)*^3^, where *λ* is the light wavelength in free space, and *n* is the material refractive index in the cavity, indicating the mode size of this device is below the diffraction limit. A *F*_*p*_ value of ~10 for our devices indicates that the spontaneous emission rate in the cavity is enhanced 10 times relative to the bulk material emission rate in a conventional diffraction limited laser cavity. The *Q* and *F*_*p*_ do not significantly change with *H* (Fig. 2c.d) for our devices, indicating that the device performance is not sensitive to the variations in the growth or fabrication.

### Waveguide coupling efficiency

**Figure 3:**
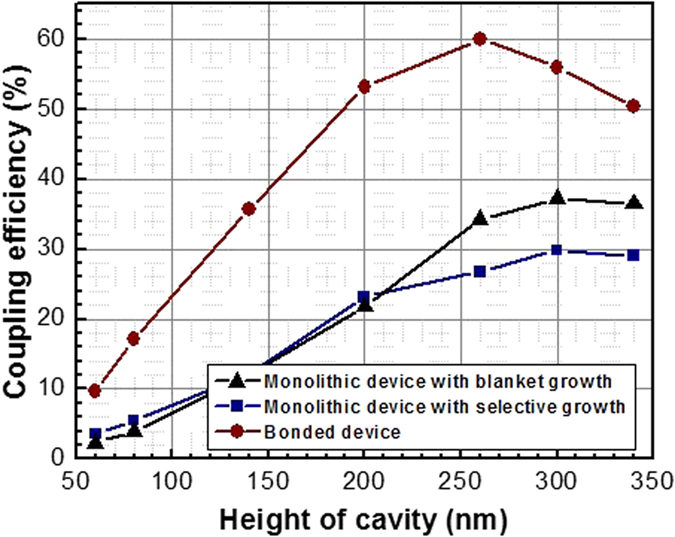
Waveguide coupling efficiency dependence on the height of the cavity for three plasmonic nanolaser configurations.

Another major challenge of monolithic lasers on Si is to achieve a high out-coupling efficiency with subsequent integration strategies. If successful, such integration allows Si-based integrated circuits and optical input/outputs (I/Os) to exist simultaneously on a single Si chip, thus enabling low parasitic capacitance, low-power consumption, and high-speed optical links^15^. In order to achieve this goal, the emission needs to be effectively coupled to the optical waveguides in a plane of the chip surface, which is part of our investigations (Fig. 3). Our analysis shows that the plasmonic mode in the cavity successfully converts emission into propagating waveguide modes beyond the confinement of the cavity region. This can be understood by light leaking into the waveguide at the edges of the cavity. Furthermore, since the 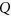-factor is inversely proportional to the radiation efficiency and the *Q* depends on the laser thickness, we observe a maximum coupling at the cavity thickness of 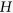
~260 nm for all cavity type investigated. For thicker devices with *H *> 260 nm, 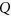 increases due to resonant mode-switching.

A maximum coupling efficiency of ~60% is recorded for the laser bonded on SiO_2_ using the Al_0.3_Ga_0.7_As/SiO_2_ waveguides through bonding. This waveguide coupling efficiency is among the highest reported to date for nanolasers and originates from our planar plasmon mode design which enables strong light coupling in the lateral instead of vertical direction. The lasers monolithically grown on Si exhibit waveguide coupling efficiency of ~35%. This light coupling efficiency can be improved by using waveguide materials with higher refractive indices and using high index-contrast cladding layers. For the rest part of the paper, all analysis is based on the devices with a height of *H *= 260 nm, corresponding to the maximum waveguide coupling efficiency (Fig. 3).

### Output power and frequency response above threshold current

**Figure 4:**
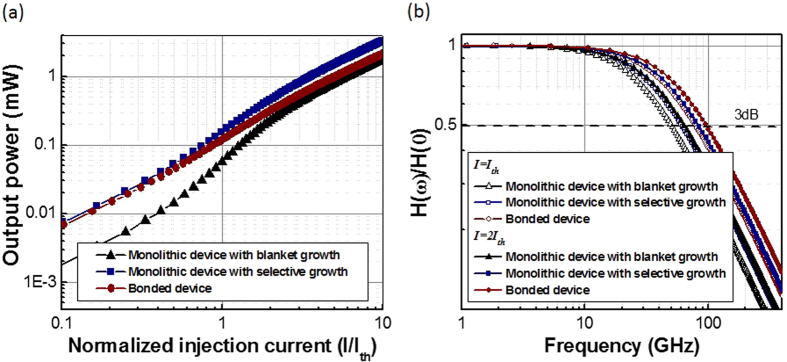
Plasmonic nanolaser performance: (**a**) Output power as a function of normalized injection current (i.e. 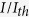), where 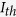 is the laser threshold current. (**b**) Frequency response at the injection current and of *I *= 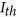 and *I = *2 × 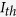.

The laser output power was evaluated using rate equations explained in the Methods section. The rate equation analysis shows that these plasmonic nanolasers have threshold currents (*I*_*th*_) of ~2.0 mA, ~2.4 mA, and ~3.1 mA, and high spontaneous emission factor *β* = 0.55, 0.48, and 0.32, for the bonded device, monolithic device with selective growth, and monolithic device with blanket growth, respectively. The laser output power versus the normalized injection current relationship (Fig. 4a) yields high spontaneous emission factor *β *= 0.55, 0.48, and 0.32 for the bonded device, monolithic device with selective growth, and monolithic device with blanket growth, respectively. These high *β* factors indicate high photon utilization of the plasmonic cavity. The power outputs shown here for the three devices are the total power emitted from the laser cavity (Fig. 4a), including the power coupled into the waveguide and the power coupled elsewhere which can also be utilized with improved waveguide design. All the devices show the comparable power output above threshold current, with the highest power obtained from the monolithic device with selective growth. For this device, the result shows that the output power at a drive current *I = *2 × *I*_*th*_ is as high as ~400 μW and ramps up quickly to >3 mW at 10 times the threshold current. These power levels are sufficient for short and mid-reach optical links.

The frequency response of the lasers was also derived from rate equations. The results (Fig. 4b) show that these nanolasers have higher modulation bandwidth than conventional bulk lasers, due to the modifications of the internal carrier lifetime, photon density, and transition rates. In addition, the modulation bandwidth increases for higher injection current due to interplay between the photonic and electronic rates of both the cavity and the external pump. Higher pump rate drives the gain medium faster into inversion. Given the high emission rate plasmonics cavity, this inversion is rapidly depleted and hence can be re-excited more promptly compared to larger diffraction limited devices. At *I = I*_*th*_, the 3 dB bandwidth of the lasers are all already beyond 50 GHz, sufficient for high-speed interconnects as directly modulated on-chip light source. At *I = *2 × *I*_*th*_, the 3 dB bandwidths are approaching 100 GHz, which is clearly superior than the conventional lasers and are very desirable for next generation high bandwidth chip I/Os.

### Output power and frequency response below threshold current

**Figure 5:**
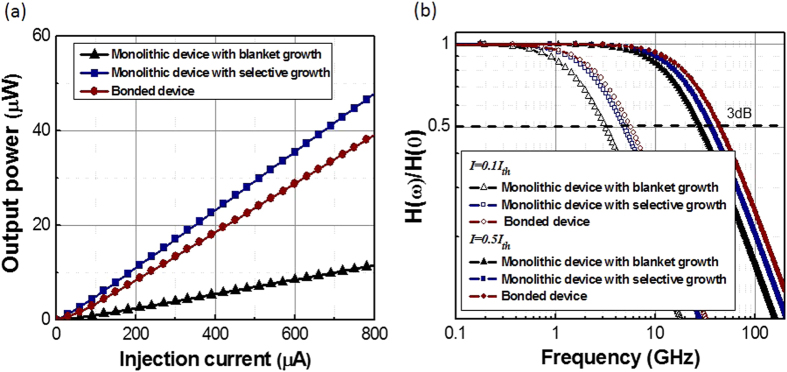
Plasmonic nanoLED performance below lasing threshold current: (**a**) Output power as a function of injection current. (**b**) Frequency response of the plasmonic nanoLED at injection current *I *= 0.1 × 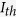 and *I *= 0.5 × 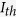, respectively.

While the output power and frequency response of these nanolasers are encouraging, the lasing threshold current density is relatively high compared to conventional lasers. This requires efficient heat sinking scheme to operate these nanolasers at room temperature continuously. Alternatively, with a lower injection current, the device can be operated below threshold conditions as a nano-cavity light-emitting-diode (LED). While the output power of such a nano-cavity LEDs (Fig. 5a) is lower than current optical interconnect sources, such devices fit well in the next generation on-chip optical interconnect applications with which predicts significant reductions in terms of energy per bit metrics^1^. The low power light detection can be achieved utilizing highly scaled plasmonic photodetectors^16^, which need less optical power to generate the same required voltage due to enhanced light-matter-interaction and reduced device capacitance. As a result, the required optical power and the total energy consumption of the optical link are greatly reduced^17^.

The frequency response of the nano-cavity LED is also of interest. Conventional LEDs have relatively low modulation speed. However, these nano-cavity LEDs have enhanced frequency response due to the Purcell effect, i.e., nano-cavity enhanced spontaneous emission rate^18^. The 3 dB bandwidth of the devices are 3~6 GHz at *I = 0.1 × I*_*th*_ and 28~44 GHz at *I = 0.5 × I*_*th*_, respectively (Fig. 5b), which is significantly faster than conventional LED^19^ devices. Given such expected bandwidths, these nano-cavity LEDs can also be used for direct modulation as on-chip light source for high-speed optical links.

### Effect of surface recombination

**Figure 6:**
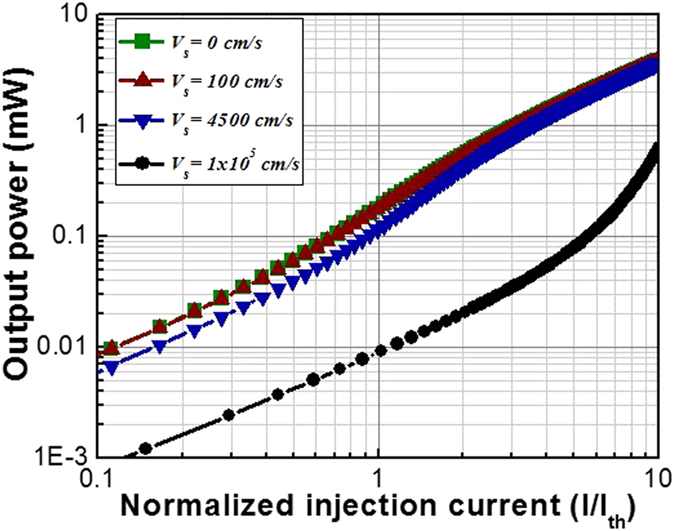
Comparison of output powers as a function of normalized injection current with various surface recombination velocities for the selective grown monolithic plasmonic nanolaser on Si.

Surface recombination is an important factor to be considered for the nano-cavity lasers due to the large surface to volume ratio of these devices. If the III–V surface on the side wall is not well passivated, the surface recombination velocity can be as high as 10^5^ cm/s. In this case the device output power will sufer serious degradation as shown in Fig. 6. On the other hand, when the surface recombination velocity is less than 100 cm/s, there is almost no effect on the device performance; this can be achieved by regrowth of the III–V passivation layers on the side walls^20^. Passivation using dielectric layers can also reduce the surface recombination velocity to ~4500 cm/s^21^. At this condition, the effect of the surface recombination is negligible (Fig. 6). The threshold current also shifts with the passivation quality. Without considering surface recombination effects, the threshold current is ~2.0 mA, ~2.4 mA, and ~3.1 mA for bonded, selective, and blanket growth devices, respectively. When the surface recombination velocity is ~4500 cm/s, the threshold current values change to ~2.14 mA, ~2.57 mA, and ~3.34 mA, respectively for the above devices.

### Effect of fabrication variations

**Figure 7:**
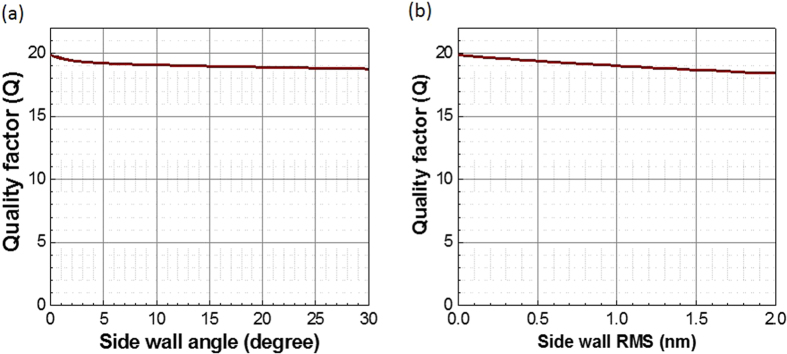
*Q* factors dependence on (a) cavity side-wall angle and (b) cavity side-wall roughness with root mean square (RMS) values.

The obtained device performance, including waveguide coupling efficiency, frequency response, and output power, are all suitable for next generation optical interconnects. However, the fabrication and integration of the nanolaser devices can be challenging, because precise fabrication steps are usually needed to minimize extra cavity losses, such as scattering due to surface roughness and radiative loss due to tilted side walls. It is important to understand how fabrication imperfections affect the device performance. We modeled the *Q* factor dependence of the nanolasers with the above two practical loss factors (Fig. 7). We find that *Q* changes less than 10% for angled side-wall up to 30^o^, which allows for even using wet chemical etching instead of dry etching to define the laser side walls. In addition, the *Q* factor only changes about 10% for a roughness from 0 to 2 nm route-mean-squares (RMS), which is a realistic and upper limit for semiconductor device processing induced roughness (Fig. 7b). The reason for this robust and insensitive performance to process variations is that most of the electric field is well concentrated near the top interface within the cavity with little field reaching the side-walls and lower part of the cavity (Fig. 2b). However, for other type of nanolasers, *Q* factor may be very sensitve to the fabrication variations. A few degree of side-wall angles may result in significant *Q* factor degradation^22^. Related to processing, this light source design appears quite tolarent to imperfections, and overall shows bennificial features for on-chip integration into interconnect links.

### Discussion

Conventional lasers that are several microns thick for edge emitting devices and close to 10 micron thick for VCSELs can not be integrated in the front end of the line (FEOL) of Si CMOS process. Whereas plasmonic nanolasers can be readily integrated at the FEOL of the Si CMOS process. The ability to further scale these devices to sizes of tens of nanometers, similar to the size of a transistor, makes the integration with CMOS even more flexible. Here the thermal budget can be controlled by fabricating the Si CMOS first with a maxium temperature of ~1000 ^o^C until before the contact silicidation step. The III–V laser material is then grown at ~650 ^o^C, which has neglegible impact on Si CMOS processes. The CMOS contact silicide process and back end of the line process temperatures are all below ~400 ^o^C and can be done after the III–V material growth.

In conclusion, we have investigated the design of III–V plasmonic nano-cavity-based light sources for monolithic integration on silicon substrates for the next generation low power consumption, highly integrated, high bandwidth optical interconnects. Due to the sub-diffraction limited laser footprint, this III–V plasmonic light source can be directly grown on silicon substrate free of crystallographic defects. Although the device has a submicron dimension, it delivers milliwatt optical power and ~100 GHz modulation bandwidth above threshold injection currents, excellent for high speed optical interconnects. When the injection current is below the threshold condition, the plasmonic enhanced nano-cavity LED exhibits tens of microwatts of output power and tens of GHz direct modulation bandwidth, sufficient for next generation low power consumption optical interconnects. It also exhibits high waveguide coupling efficiency to conventional optical waveguide and exellent compatibility with Si CMOS process. The device performance is robust and tolerant to fabrication imperfections. Overall, this design provides a practical and advantageous route to monolithically integrated nanoscale light sources on silicon substrates for optical interconnect and other applications.

## Methods

### FDTD optical simulation

The optical design of the nanolasers is performed using commercially available finite difference time domain (FDTD) software (Lumerical Solutions, Inc.). The input of complex refractive indices (i.e. 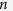
and 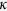) such as Gold, GaAs, and SiO_2_ are taken from the built-in material database. For the alloys of the utilized III–V material (Al_x_Ga_1-x_As) data from ref. 23 is used for the dispersion relation according to the Kramers-Kronig relation. Based on this dispersion data an analytic fitting function (multi-coefficient material model) generates the required index data in the bandwidth of interest. In order to provide a plasmon excitation, randomly placed vertically oriented (i.e. 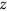-direction) electric dipole sources are placed inside each cavity to excite the cavity resonance. Here, the 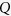-factor is calculated from the Fourier transform of the electromagnetic field by finding the resonance frequencies (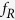) of the signal and measuring the full width at half maximum of the resonant peaks (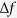), i.e. 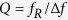. A 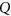-analysis option within the solver is utilized to obtain the 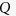
factors, and the corresponding resonant wavelength (i.e.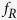) can be found from each resonant mode. The dipole excitation source used in FDTD Solutions allows to return the Purcell factor as a result that can be directly visualized, which is equivalent to dividing the power emitted by a dipole source in the cavity environment by that of a homogeneous bulk material.

### Rate equation simulation

We evaluate the device performance parameters relating to output power and modulation speed using laser rate equations (1) and (2)^24^. In order to relate the rate equations to the power output, 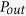, can be written as in equation (3)^25^. The small signal response of the plasmon laser can be expressed as equation (4) by observing the spectral response function^26^.


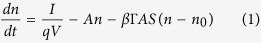



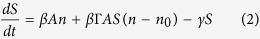



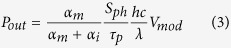



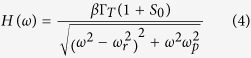


where 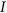 is the injection current, 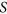 is the photon number of a single lasing mode, 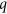 is the elementary charge, 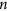 is the excited state population density, 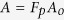 is the spontaneous emission rate, 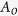 is the natural spontaneous emission rate of the material, *β* is the spontaneous emission factor, 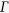 is the overlap factor, 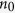 is the excited state population at transparency, 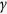 is the total cavity loss rate per unit volume, 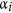 is the cavity intrinsic loss per unit length, 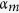 is the cavity mirror loss per unit length,
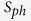
is the photon density, 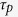
is the photon life time, and is proportional to the cavity 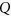 (i.e.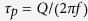, 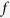 is the cavity resonant frequency), 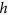 is the planck constant, 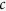 is the light speed in vacuum, 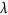 is the lasing wavelength, 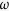 is the optical cavity angular frequency, 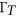 is the transition rate of excited state population, 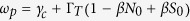, 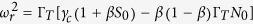, 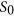 and 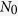 are the steady-state photon number and population inversion number, respectively, and 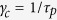.

When surface recombination is taken into account, the rate equation (1) is written as:


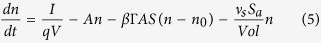


where *v*_*s*_ is the surface recombination velocity, *S*_*a*_ is the surface area, and *Vol* is the volume of the cavity.

## Additional Information

**How to cite this article**: Li, N. *et al.* Monolithic III–V on Silicon Plasmonic Nanolaser Structure for Optical Interconnects. *Sci. Rep.* **5**, 14067; doi: 10.1038/srep14067 (2015).
